# Genotypic and phenotypic character of Chinese neonates with congenital protein C deficiency: a case report and literature review

**DOI:** 10.1186/s12959-019-0208-6

**Published:** 2019-10-02

**Authors:** Xiaoying Li, Xiaoyan Li, Xiao Li, Yuanhua Zhuang, Lili Kang, Xiuli Ju

**Affiliations:** 1 Qilu Children’s Hospital of Shandong University, Jinan, Shandong China; 2 People’s Hospital of Rongcheng, Weihai, Shandong China; 3 Qilu Hospital of Shandong University, No107, Cultural west Road, Lixia District, Jinan, Shandong China

**Keywords:** Neonate, *PROC* gene, Protein C, Purpura fulminans, Thrombosis

## Abstract

**Background:**

Our objective was to study the phenotype of and molecular genetic mechanisms underlying congenital protein C (PC) deficiency in Chinese neonates. We report the case of a neonate who presented 4 h after birth with purpura fulminans of the skin and thrombosis in the kidney. We also carried out a through literature review to study the genotype and phenotype, relevance, diagnosis, management, and prognosis of neonates with congenital PC deficiency in China.

**Case presentation and literature review:**

Following a septic work-up and check of PC and protein S (PS) levels that showed PC deficiency, we investigated the patient’s and her parents’ genotypes. Our patient was found to have a plasma PC level of 0.8%. Molecular testing revealed a compound heterozygous mutation of the *PROC* gene: From the father, a c._262 G > T p. ASP88Tyr mutation in exon 4; from the mother, a C. 400 + 5G mutation in intron 5 that had been previously reported as likely pathogenic. Both parents were found to have heterozygous mutations for PC deficiency. In China, 5 other cases of congenital PC deficiency in the neonatal period were reported in the literature. In those cases, purpura fulminans and thrombosis were the main symptoms, and homozygous or compound heterozygous mutations of the *PROC* gene were identified.

**Conclusion:**

Congenital PC deficiency should be ruled out for neonates presenting with purpura fulminans and thrombosis.

## Background

Congenital protein C (PC) deficiency is a rare genetic disorder associated with an elevated risk of venous thromboembolism (VTE) and an incidence of 1/40,000–1/250,000 [1–3]. Patients with a mild form of this disorder may be asymptomatic but remain susceptible to VTE at any age [4]. Severe congenital PC deficiency is a life-threatening coagulopathy associated with significant clinical signs of thrombophilia, including purpura fulminans and substantial skin necrosis during the early neonatal period. Protein C is encoded by the *PROC* gene on chromosome 2q13–q14, which consists of nine exons (1790 bp) [5]. Congenital PC deficiency is typically inherited in an autosomal dominant manner from heterozygous carriers. Severe PC deficiency has been associated with homozygosity or compound heterozygosity for *PROC* gene mutations that affect the normal sequence of mRNA and the protein synthesis [4]. Here, we report a neonate who presented with purpura fulminans on the first day of life. We discuss the clinical phenotype and the localization of the genetic mutations. We also present a comprehensive review of all cases of neonatal congenital PC deficiency reported in China during the past 40 years.

## Case presentation

**Fig. 1:**
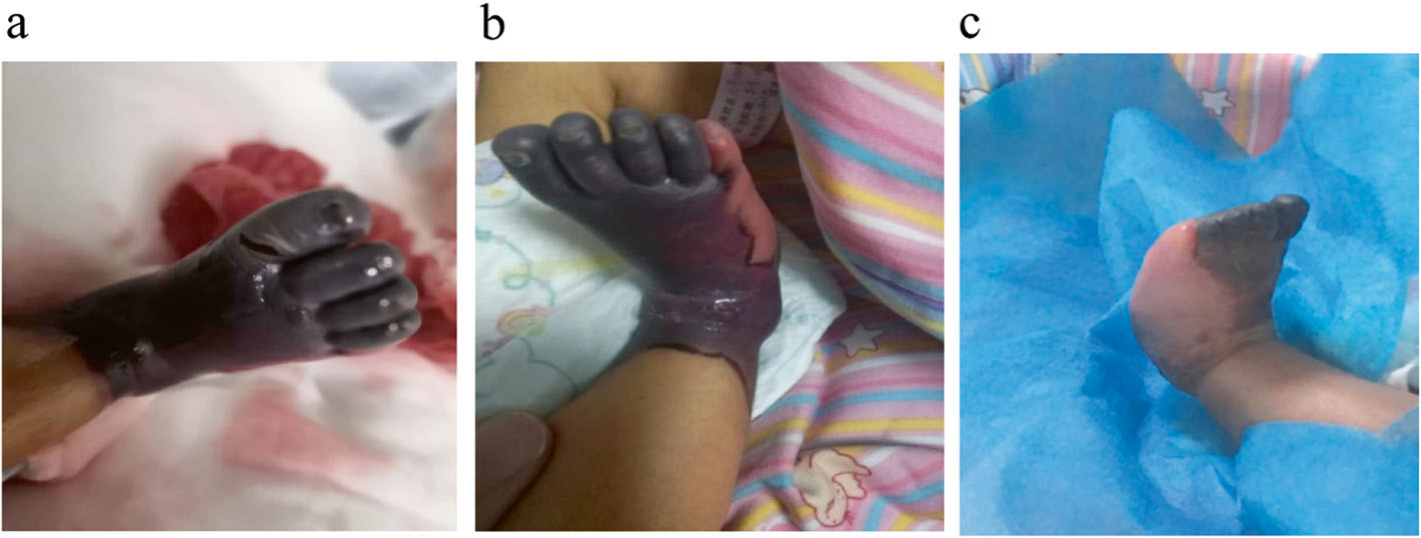
Phenotype of a One-day-old Female Presenting with Purpura Fulminans. A one-day-old female neonate was transferred to our neonatal intensive care unit from a local hospital presenting with purpura fulminans that began four hours after birth. Note the progressive necrotic lesions from toe to heel on the right foot (**a**–**c**)

A one-day-old female neonate was transferred to our neonatal intensive care unit from a local hospital with a main complaint of progressive necrotic lesions from toe to heel on the right foot beginning at four hours after birth (Fig. 1). The patient was a G2P2, small-for-gestational-age neonate with birth weight 2.36 Kg and gestational age 38 weeks. She had a complicated perinatal history of fetal distress and premature rupture of membranes (PROM) with blood-stained amniotic fluid. Before birth, fetal ultrasound revealed a left kidney enlargement. At birth, the patient experienced an episode of asphyxia and required intensive resuscitation. She had an Apgar score of 1′-4, 5′-7. The patient’s physical examination was generally unremarkable except for the black, necrotic skin lesions covering the right foot. The maternal history was unrevealing, and the mother had one other child: a healthy, four-year-old boy.

**Fig. 2:**
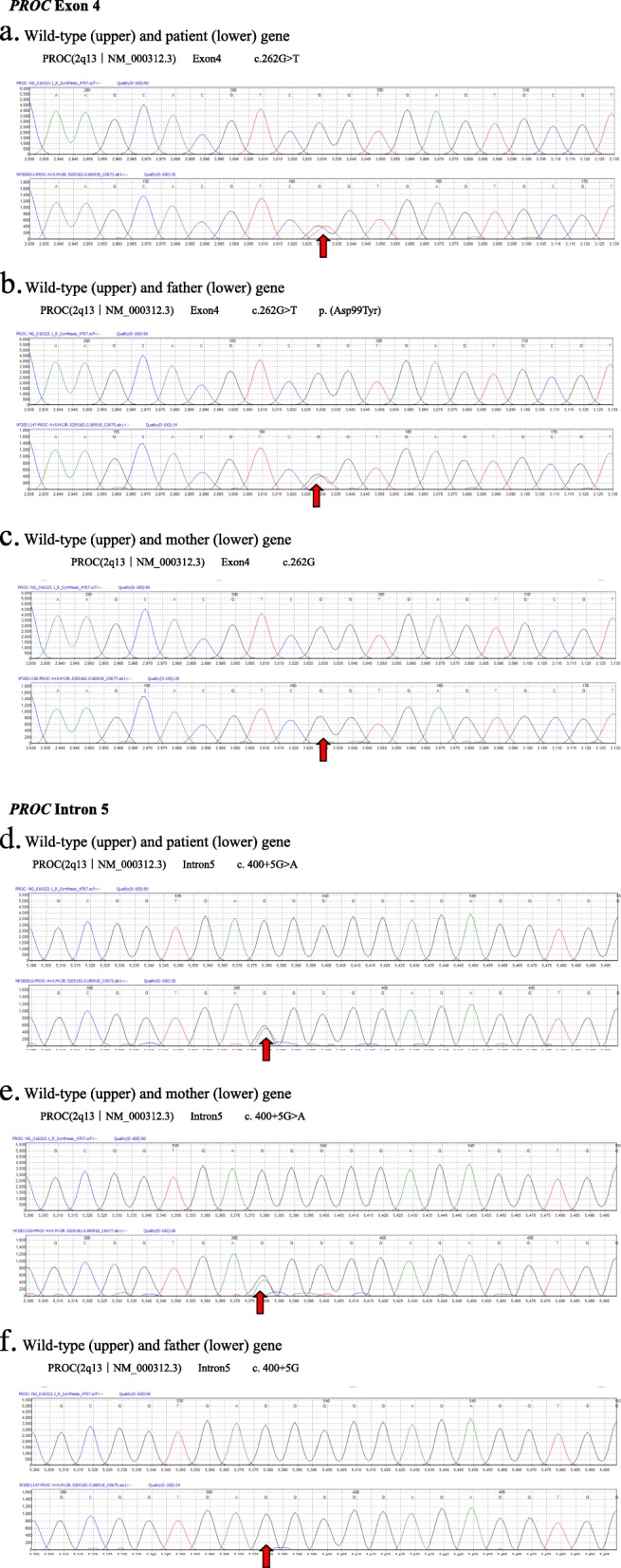
Sequence Diagrams for the Patient and Parents. A genetic evaluation revealed the patient had compound heterozygous mutations of the *PROC* gene (NM-000312.3) inherited from the father and mother individually. From the father, a c._262 G > T mutation in exon 4 (red arrows in **a**–**c**). From the mother, a c.400 + 5G > A micromutation in intron 5 (red arrows in **d**–**f**). Both parents were heterozygous carriers for the mutation individually

Antibiotics were started initially for suspected sepsis and vitamin K was given at the first admission day. After a clinical diagnosis of congenital PC deficiency was made, fresh frozen plasma (FFP) was given at 10 mL/kg per day following injection of low-molecular-weight heparin (LMWH). A laboratory work-up was carried out and PC activity levels were measured with a chromogenic assay. Results showed the following: red blood cell count (RBC): 3.3 × 10^12^/L; hemoglobin (Hb): 11.2 g/dL; Hct: 32.7%; MCV: 99.2 fl; WBC: 13.00 × 10^9^/L; platelet: 299 × 10^9^/L; PT: 17 s; PTT: 49.1 s; PT-INR: 1.38; D-dimer: 24.04 mg/dL; fibrinogen: 1.29 mg/dL. The patient’s plasma PC activity level was severely low at 0.8% (reference range 70–140%). Protein S (PS) level was 90.7% (reference range 60–130%), and lupus anticoagulant (LA) factor ratio LA1/LA2 was 0.96 (reference range 0.8–1.2). An ultrasound showed a left kidney infarction. Both the patient’s parents were found to have low PC activity (mother: 48.7%; father: 64.7%) and normal PS activity (mother: 63.6%; father: 80.7%) (Table 1). We carried out a genetic evaluation of our patient and both her parents. This investigation revealed two heterozygous mutations of the *PROC* gene (NM-000312.3) inherited from the father and mother individually. From the father, a c._262 G > T mutation in exon 4 that had previously been reported as pathogenic. From the mother, a c.400 + 5G > A micromutation in intron 5 that we believe was pathogenic and caused the low PC activity level in the mother, and had previously been reported in PC deficiency patients as likely pathogenic. Both parents were found to be heterozygous carriers for the mutation individually (Fig. 2).

**Table 1:** Selected Laboratory Results for the Patient and Parents

Measure (units) (Reference range)	PC (%) (70–140%)	PS (%) (60–130%)	PLT (×10^9^/L)	D-dimer (mg/L) (0-1 mg/L)	Fibrinogen (g/L) (2-4 g/L)
Patient	0.8	90.7	299	24.04	1.29
Mother	48.7	63.6	287	0.5	3.2
Father	64.7	80.7	319	0.4	3.5

Abbreviations: *PC* protein C, *PLT* platelet count, *PS* protein S

Our patient remained stable during her hospitalization except for the necrotic skin lesions. Her vital signs also remained stable. The skin lesions on her foot resolved under the FFP and LMWH SC treatment. The patient was not treated with activated PC concentrate because this product is currently not available in China. She experienced another two episodes of purpura fulminans on her left arm and abdomen wall in the hospital. Repeat assessments showed her plasma PC activity level remained stable at 0.8% with no improvement. Our patient’s total hospital admission time was 50 days. During this time, her skin lesions resolved. Subsequently, her enlarged kidney reduced in size and she recovered from the left kidney infarction. Our patient passed away two months after presentation.

## Discussion and conclusions

Protein C is a vitamin K-dependent plasma glycoprotein zymogen synthesized in hepatocytes [6] and activated by the thrombin-thrombomodulin complex. It exerts anticoagulant activity through inactivation of Factor V and VIII via cleavage of bonds of the active forms of these factors [6, 7]. Protein S, Factor V, calcium, and phospholipids are co-factors involved during this process [8]. Low plasma PC levels increase the risk of thromboembolic events. Congenital PC deficiency is a rare cause of hereditary thrombophilia and is implicated in only about 3–5% of all thromboembolic events [1]. In the adult Asian population, studies from Taiwan, Hong Kong, Japan, and Thailand concluded that thrombophilia is associated with PC system dysfunction via various rare mutations [9–12]. Recently, Tang L et al. completed a case-control study of 310 adult VTE patients showing that the most common genetic risk factor for VTE in the Chinese population, with a genetic prevalence of approximately 2.36%, is a PROC c.574_576 del variant associated with both decreased PC anticoagulant activity and increased risk of VTE [13]. Thrombotic manifestations are relatively rare (less than 5%) in children with inherited thrombophilia [14]. Fewer reports are published in this area.

PC deficiency was first described in 1962 as a suspected inherited lesion identified in three siblings with similar skin lesions [5]. In 1976, Stenflo [4] first described a vitamin K-dependent anticoagulant glycoprotein synthesized by the liver as a proenzyme for PC deficiency. PC deficiency is very rare, with a frequency of 1 case in 40,000–250,000 individuals [15]. The frequency of homozygous PC deficiency is estimated at 1 in 500,000–750,000 neonates, with an equal distribution of males and females [16]. The typical clinical symptoms of PC deficiency are purpura fulminans, disseminated intravascular coagulation, and thrombotic complications or subsequent hemorrhage during the neonatal period. The plasma PC level of the neonate is physiologically low and reaches adult levels by six months of age [17]. A physiologically low vitamin K status after birth further increases the risk of mortality related to low PC activity during the neonatal period. Thus, neonates with congenital PC deficiency typically exhibit severe symptoms and high mortality rates. A search of the literature reveals that almost 30 cases of neonatal congenital PC deficiency were reported globally. In mainland China, 5 cases of neonatal congenital PC deficiency were reported during the past 40 years.

There are two types of congenital PC deficiency: type I (quantitative) is characterized by reduced PC antigenic level and function; type II (qualitative) [18] is characterized by decreased PC function relative to the antigenic level. The majority of PC deficiency cases are of type I [7].. In our patient’s case, the type of deficiency (I or II) was unknown since PC activity levels, but not antigen levels, were measured. Congenital PC deficiency is encoded by the *PROC* gene on the long arm of chromosome 2q13–q14, which consists of nine exons (1790 bp). Mutations in the *PROC* gene cause PC deficiency, which is inherited in an autosomal dominant manner (leading to the heterozygous form) or an autosomal recessive manner (leading to the homozygous form). More than 360 unique mutations in the *PROC* gene have been identified to-date [7]. Most of these are missense or nonsense mutations that cause either quantitative PC deficiency (type I) or qualitative PC deficiency (type II). In adults, heterozygous PC deficiency is associated with a 10-fold increase in the risk of developing thromboembolic events compared to the general population [19]. Although homozygous or compound heterozygous PC deficiencies usually lead to life-threatening thrombosis and increased mortality rates in neonates [20], previous reports indicate that some homozygous PC-deficient patients may have a milder course with low but measurable PC levels [21]. One such patient who had a PC level < 10% was reported to be asymptomatic at the age of 38 years [22]. This suggests that the course of this disorder may vary in patients with relatively higher PC levels. Of note, other reports showed family histories of third-degree consanguineous marriage for some patients with PC deficiency [23, 24].

Our patient was compound heterozygous for PC deficiency, with one *PROC* gene mutation on exon 4 inherited from the father and the other mutation on intron 5 inherited from the mother. The compound heterozygous *PROC* exon gene mutation caused the low plasma PC level of 0.8%. Her high D-dimer level and low fibrinogen level revealed thrombosis was ongoing. The severe clinical consequences of the patient’s extremely low PC level (0.8%) caused thrombosis in the left kidney during the fetal period and purpura fulminans with hallmark necrotic skin lesion soon after birth. Her parents both had differing low levels of PC and normal levels of PS, Di-dimer, and fibrinogen without clinical symptoms. We hypothesize that both the severely low fetal PC level and the moderately low maternal PC level related to the maternal heterozygous *PROC* gene intron mutation may have resulted in the PROM and blood-stained amniotic fluid recorded in the perinatal history by causing abnormal coagulation leading to thrombosis and hemorrhage in the placenta.

Table 2 presents a summary of key baseline, clinical, physiological (PC), and genetic characteristics of the 6 neonatal congenital PC deficiency cases reported in China during the past 40 years [8, 25–27]. These patients include our current case and 5 others reported in the literature. Among reported cases, the ratio of males:females was even at 3:3, even though some reports suggest morbidity is higher males than in females. One patient was late preterm and 5 were term at birth, including 1 intrauterine growth restriction. All showed typical symptoms within 2 days after birth. All had significant symptoms that included purpura fulminans, cranial thrombosis (6 patients), eye injures (2 patients), intracranial hemorrhage (2 patients), or renal thrombosis (2 patients). The earliest-reported case was from 1988. That case was based on a clinical diagnosis without plasma PC level or genotype. In all 5 cases with PC levels available, patients had PC levels ranging from < 1–22% (normal range: 60% or 70–140%). Four cases also had severely low PC levels ≤8%. The lowest PC level was 0.8% (in our patient), and one case had a PC level of 22% (almost 1/3 of normal) and presented with skin lesions. M. Ichiyama and colleagues reported a Japanese deep vein thrombosis patient with a similar PC level of 21% who presented with fetal hydrocephalus and neonatal stroke [28]. The authors detected a heterozygous c.574_576delAAG, p.Lys193del mutation in exon 7 of the *PROC* gene. The 3-base deletion raises the thromboembolic risk in Japanese and Chinese populations [13]. Two cases reported before 2016 were diagnosed based on PC level alone (without genotype data). Of the 3 cases with genotype available, all had compound heterozygous *PROC* mutations. Where PC levels of the parents were measured, all were mildly lower than the normal range and not symptomatic.

**Table 2:** Summary of Clinical Characteristics and Laboratory Results for Neonatal Congenital Protein C Deficiency Cases Reported during the Past 40 Years in China

Case	Year	Sex	Age	GA (wk)	BW	Clinical Symptom			PC Level (%) (ref: 70~140%) Patient Mother Father			Mutation
						Skin	Thromb	Hemorrhage				
1	1988	male	19 h	term	–	yes	–	cranial	no	no	no	not investigated
2	2012	male	10 h	39	–	yes	–	no	22	61	53	not investigated
3	2015	female	38 h	35^+ 6^	2300	yes	cerebral eyes	cranial	8.0^a^	51^a^	60^a^	not investigated
4	2015	female	1 day	39^+ 6^	2780	yes	cerebral	no	< 1	49	53	c.755C > T(P. 252.A > V)^c^ intron5 + 5G > A^d^
5	2017	male	1 day	term	–	yes	yes	no	4	43	50	exon8(c.795_796insA)^c^ exon9(c.1206_1207insG)^d^
6^b^	2018	female	43 h	34	2300	yes	yes	no	0.8	48.7	64.7	exon4 c.262G > T p. (Asp99Tyr)^c^ intron5c.400 + 5G > A P?^d^

^a^Reference range 60–140%

^b^ Denotes our current case

^c^ Mutation inherited from father

^d^Mutation inherited from mother

Abbreviations: *BW* birth weight, *GA* gestational age, *PC* protein C, *ref*., reference range, *Thromb* thrombosis, *wk*. weeks

For the parents of neonates with congenital PC deficiency, molecular diagnosis offers an option for a reliable prenatal diagnosis during the next pregnancy. Early diagnosis can be especially important considering the poor prognosis for this serious condition and the associated need for multidisciplinary care to address the ophthalmological and neurological sequelae in affected individuals.

The suggested treatment approach for PC deficiency is PC replacement using FFP, activated PC concentrate, or human PC concentrate. After bleeding is controlled, anticoagulants can be initiated; LMWH is recommended. The American College of Chest Physicians (ACCP) guidelines for antithrombotic therapy in symptomatic neonates and children recommend treatment with either 10 to 20 mL/kg FFP every 12 h or PC concentrates at 20 to 60 IU/kg until resolution of clinical lesions. Prophylactic treatment is initiated after stabilization of the clinical symptoms. PC concentrate may be individually reduced to a prophylactic level: Dose regimens of 24 to 90 IU/kg once a day, 250 to 350 IU/kg every other day, or 90 IU/kg three times a week have been reported [19]. A curative therapy for severe congenital PC deficiency is liver transplantation. We treated our patient initially with FFP replacement, and then with LMWH by subcutaneous injection. We did not give activated PC concentrate because the PC product is currently not available in China. At discharge, she was receiving only LMWH injections.

For neonates with PC deficiency, the prognosis is poor, and the mortality rate is higher than for adults with the condition. Neonates who survive infancy are at risk for poor neurological outcomes due to clinical symptoms such as intracranial thrombosis and hemorrhage. In all 6 congenital PC deficiency cases reported in China, the infants received no comprehensive follow-up care and subsequently died. In conclusion, congenital PC deficiency, although rare, should be considered in neonates presenting with purpura fulminans and thrombosis. Early diagnosis and replacement of PC deficiency may prevent or reduce the mortality and severe morbidities typically associated with this condition. In all cases we reviewed (our own case and the other 5 cases reported in China), the neonates presented with extremely low PC levels and severe clinical signs, and experienced early death. We identified that compound heterozygous *PROC* gene mutations inherited separately from the father and mother can be responsible for the severe phenotypes of these patients. We recommend further family pedigree study be done for the family health consultation when a neonate presents with possible PC deficiency.

## Data Availability

Data sharing is not applicable to this article as no datasets were generated or analysed during the current study.

## Abbreviations

ACCP: American College of Chest Physicians
FFP: Fresh frozen plasma
LMWH: Low-molecular-weight heparin
PC: Protein C
PROM: Premature rupture of membranes
PS: Protein S
VTE: Venous thromboembolism
